# Pediatric Obstructive Sleep Apnea Syndrome: Emerging Evidence and Treatment Approach

**DOI:** 10.1155/2021/5591251

**Published:** 2021-04-23

**Authors:** Maria Rita Giuca, Elisabetta Carli, Lisa Lardani, Marco Pasini, Marco Miceli, Eleonora Fambrini

**Affiliations:** Department of Surgical, Medical and Molecular Pathology and Critical Area, Dental Clinic, University of Pisa, Pisa, Italy

## Abstract

OSA pediatric subjects suffer from episodes of upper airway obstruction that can be partial or complete, with atypical sleep patterns and blood-gas level alteration. If poor treated and/or diagnosed, it can cause cardiovascular disease, learning difficulties, behavioural issues, and retardation of growth. In the literature, there are conflicting evidence about OSA assessment and treatment in pediatric age, so the aim of this paper is to highlight the multidisciplinary approach in the management of sleep disorders, stressing the role of the pediatric dentist in both diagnosing and treating the OSAS in children, according to the current evidence of the treatment options effectiveness of the syndrome itself. *Conclusions*. Scientific evidence shows that OSAS management requires a multidisciplinary approach in order to make an early diagnosis and a correct treatment plan. The orthodontic treatment approach includes orthopedic maxillary expansion and mandibular advancement using intraoral appliances. Hence, the orthodontist and the pediatric dentist play an important role not only in early diagnosis but also in the treatment of pediatric OSAS.

## 1. Introduction

Sleep-disordered breathing (SDB) in children consists in a wide spectrum of respiratory disorders, which are commonly characterized by upper airway increased resistance, with pulmonary ventilation temporary interruption and sleep quality alteration [1]. SDB clinically related conditions vary from simple snoring to more complex manifestation, such as the Obstructive Sleep Apnea Syndrome (OSAS). OSA pediatric subjects suffer from episodes of upper airway obstruction that can be partial or complete, with atypical sleep patterns and blood-gas level alteration. In particular, Obstructive Sleep Apnea Syndrome (OSAS) in children can cause cardiovascular disease, learning difficulties, behavioural issues, and growth retardation, if poorly treated and/or diagnosed [2].

OSAS specific pathogenic mechanism results during sleep in a condition of hypoventilation associated with pharyngeal muscles physiological hypotonia, together with a nonphysiological oropharyngeal space reduction, nasal resistance increase, tongue retroposition, and upper airway shrinking [3].

While in adult patients it can be detected nonpathological OSA episodes, in children every apneic episode must be considered pathological, as in growing patients, the physiological upper airway resistance increase during sleep is moderate and so unable to establish apneic episodes [2–4].

However, SDB is commonplace in pediatric patients and it is induced by neurophysiological changes, caused by pharyngeal walls muscle tone alteration. The most common reasons why the muscle tone variations are established are associated with tonsillar and/or adenoidal hyperplasia.

SDB etiopathogenesis in children can be studied by age: in younger subjects, the main causes are dealing with anatomical and neurofunctional issues (nasal obstruction, neuromuscular variations, soft tissue compromising, and reduced skeletal growth) while older subjects show obesity as SDB leading cause [3].

As SDB can be considered a continuum of breathing disorders, it can be possible to distinguish, in increasing severity order: (a) primary snoring, (b) UARS (Upper Airway Resistance Syndrome), (c) obstructive hypoventilation, and finally (d) OSAS.

- Simple snoring: less severe and most common condition (occurs in 3–15% of the pediatric population, especially between 3 and 6 years (13–35%)), characterized by upper airway partial obstruction clinically manifested by soft palate vibratory noises; often associated with OSA and UARS. Simple snoring is not associated with abnormal blood-gas levels and fragmentation and/or deconstruction of sleep. Main causes: obesity, respiratory infections, adenotonsillar hypertrophy, and nasal obstructions.
- UARS: represented by during sleep increased efforts in breathing muscles activity, caused by higher upper airway resistance and negative endoesophageal pressure, which are associated with poor sleep quality and frequent arousals. Moreover, daytime irritability, reduced weight increase, and poor school performance are clinically evident. Another clinical consequence is the reduction in height development, caused by growth hormone (GH) secretion decrease, which physiologically should occur during sleep. UARS diagnosis is performed by PSG (polysomnography) [2].
- Obstructive hypoventilation: consisting of a condition of prolonged hypoxia and hypercapnia and diverging from OSAS for the absence of a complete airway cyclic obstruction (due to the different activation pattern of the pharyngeal dilator muscles, in children, which can prevent complete airway collapse) and a reduced alteration of sleep structure and quality [5, 6].
- OSAS: with a prevalence of 1–5% among the pediatric population and a peak incidence between 2 and 6 years, it is clinically characterized by prolonged upper airway partial and total obstruction. The airflow reduction (hypopnea or cessation) is defined as apnea and establishes itself together with narrowing among the pharyngeal space up to complete pharyngeal walls collapse. Pediatric OSA episodes last more than 5 seconds, with up to 4% oxyhaemoglobin reduction, hypercapnia, awakenings, and abdominal and/or thoracic breathing movements. Any breathing cessation during sleep is considered apnea in children, regardless of its duration (OSA = apnea/hypopnea index (AHI) ≥ 1). The pediatric OSAS aetiology recognizes adenotonsillar hypertrophy as the main cause of upper airway obstruction, with a maximum incidence peak between 2 and 8 years, when a wider volume is occupied by adenotonsillar tissue [2].

The clinical evaluation of SDB pediatric subjects consists in a first study of medical history, clinical exam, and eventually instrumental assessment, too.

OSA patients show a wide variability of breathing disorders in-sleep manifestations, which can strongly impact on children life quality, resulting in diurnal and nocturnal symptoms (Table 1).

The diagnosis flowchart of pediatric OSAS includes different evaluation and instrumental analysis:

- In the literature are reported several different questionnaires bound to investigate OSAS history. In particular, for what concerns OSAS in the pediatric age, Chervin designed the Pediatric Sleep Questionnaire [7], with a short version of 22 questions. Other important screening instruments are provided by the I'M SLEEPY questionnaire and by the more recent pediatric adaptation of the ESS (Epworth Sleepiness Scale) [8, 9].
- The OSAS clinical evaluation starts with the ENT exam (Ear Nose and Throat). It is an objective clinical examination that studies and grades the tonsillar hypertrophy presence, according to the Brodsky scale or the Friedman modified Mallampati classification: the first one quantifies the oropharynx volume percentage occupied by tonsillar tissue; while the second one assesses the airway obstruction grade induced by tongue [10]. The ENT exam is the greater exemplification of the multidisciplinary approach in OSAS diagnosis and/or assessment and of the needed collaboration between the ENT specialist and the pediatric dentist. In fact, the ENT exam should also study the skeletal class, if orthognathic, retrognathic, or prognathic, and the maxillary shape (ogival palate) and facies. Moreover, assessment of BMI and weight growth curve, measurement of blood pressure, and exclusion of any signs of pulmonary hypertension should be performed.
- Endoscopic evaluation: controversial for OSAS diagnosis in pediatric patients [11].
- Polysomnography: representing the gold standard in diagnosing pediatric OSAS. Polysomnography's (PSG) aim is diagnosing, differentiating, and quantifying obstructive, mixed, and central apnea and to identify and classify hypopneas, high resistance syndromes, and sleep fragmentation. At least two complete night-time sleep cycles should be covered by PSG, in a nonsleep deprivation or premedication condition. PSG is performed in pediatric age with recordings of 11–12 hours in preschool patients and of 9–10 hours in school-age subjects. PSG is a very expensive exam, requiring special equipment, room, and personnel. A detected airflow reduction greater than 90% and lasting two respiratory circles or more is considered apnea while an airflow reduction ≥ 30% lasting two respiratory circles or more is defined as hypopnea. When PSG is performed in children, every single apnea or hypopnea episode detected per hour has to be considered pathological. Moreover, according to AHI, PSG allows identifying 3 severity degrees: mild OSAS (AHI 1–4), moderate OSAS (AHI 5–9), and severe OSAS (AHI ≥ 10) [12].
- Night pulse-oximetry represents a good screening tool, with 97% predictivity, low cost, and easy applicability [2].

Despite the previously described tools and flowcharts, sleep-disordered breathing assessment and management are often complex in children due to the variety and controversy in evidence about diagnosis and treatment choices effectiveness in growing patients. The aim of this paper is to provide an update in OSAS diagnosis and therapy state-of-the-art and to stress the key role of the pediatric dentist in the diagnosis and treatment of OSAS in children.

## 2. The Role of the Pediatric Dentist

Children suffering from SDB should be managed by a multidisciplinary team consisting of a pediatrician, an ENT specialist, a pediatric dentist/orthodontist, and a speech therapist.

In particular, the pediatric dentist plays a major role, together with the otolaryngologist, in the pediatric OSAS detection, because most of the time he is the first doctor that visits the child, clinically notifying risk factors which can define the subject as suspected for apneic episodes. By the way, all the already diagnosed OSAS children should be referred to the pediatric dentist too, in order to have an evaluation of the dental-related factors, that are connected with the presence of apneic episodes done and so establish a cause-guided treatment.

It is important also to stress that, for pediatric OSAS, the diagnosis of certainty comes only from the otolaryngologist with PSG.

Therefore, the pediatric dentist can be considered as a sentinel in the individuation of pediatric subjects that can develop OSAS or already suffer from this syndrome.

Hence, it is fundamental to underline the greater *risk factors* for OSAS in growing patients, with greater attention towards the dental-related factors:

- Adenotonsillar hypertrophy (primary cause): main issue correlated with OSA in children. The evidence shows that the success rate of an adenotonsillectomy intervention in OSA therapy and resolution is about 83% in children [13].
- Obesity (especially in adolescence), insulin resistance, hepatic steatosis, and increased production of proinflammatory mediators, that is, leptin.
- Abnormalities of the maxillary structure (micrognathism and retrognathism) or of the soft tissues (macroglossia) can contribute to the reduction of the oropharyngeal space and the increase of nasal resistance. Dentofacial anomalies are frequent in children with OSAS, proving to be a consequence of OSAS, as well as a cause of apnea itself.
- Neuromuscular disorders, which lead to insufficient control of central and peripheral airflow, causing an increased tendency to the collapse of the pharyngeal-hypopharyngeal walls and a muscle tone reduction.
- Obstructive nasal diseases, such as asthma or allergic rhinitis, are pediatric determinants of nasal congestion.
- Other factors: age, family history, craniofacial dysmorphism, syndromic diseases (Down syndrome or Pierre Robin sequence), prematurity or multiple pregnancies, environmental exposure to smoke [2].

The current available symptomatic or causative *treatments* for children suffering from OSAS include the following [14]:

- Lifestyle changes–in particular, weight loss in obese subjects
- Continuous positive airway pressure (CPAP): while the literature reports CPAP to be the golden standard treatment of OSAS in adults, in the pediatric population, it has been reported as useful only in (i) patients ineligible for surgery, (ii) patients awaiting surgery, (iii) patients with persistent disease after surgery, and (iv) patients with other diseases (i.e., Down syndrome and craniofacial anomalies). It is considered as a palliative treatment that causes discomfort in children, which mostly do not get used to and so become uncooperative [15].
- Pharmacological agents.
- Surgery (especially adenotonsillectomy).
- Orthodontic treatments:
- RME (rapid maxillary expansion).
- Myofunctional devices.
- Mandibular advancement devices (MADs).

Thus, the pediatric dentist plays a key role in OSAS treatment by the use of appropriate orthodontic appliances.

The literature remarks that an association does exist between alteration in maxillary structure growth and OSAS in children, with a bilateral cause-effect relationship.

Indeed, a pathological chronic protracted respiratory pattern in childhood is associated with skeleton and soft tissue anomalies, which identify a particular cephalometric stereotype: hyperdivergence, maxillary contraction, upper and lower dental arches crowding, and narrow palatal vault [3].

Moreover, according to recent studies [16], it was demonstrated that the specific cephalometric stereotype associated with respiratory dysfunction is varying depending on the obstructive tissue involved:

- Adenoid hypertrophy subject is mainly characterized by a dolichofacial typology.
- Tonsillar hypertrophy child is mainly associated with a horizontal mandibular growth tendency, together with the tendency of a counterclockwise mandibular rotation [17].

In addition, growing patients affected by OSAS can be divided into two classes for clinical craniofacial characteristics: mouth breathing and nonmouth breathing subjects.

- *Mouth breathing* patients are characterized by a hyperdivergent facial pattern, postrotation of the lower jaw plane, and upper airway space reduction. Furthermore, unilateral or bilateral crossbite is often found [3, 18].
- *Nonmouth breathing* children exhibit II skeletal class with low mandibular growth, retracted tongue position, and deep bite [1, 3].

By the clinical evaluation, a major classification between two OSAS phenotypes emerges:

- *Classic* OSAS phenotype (presence of adenotonsillar hypertrophy in children, with or without malocclusion)
- *Congenital* OSAS phenotype (craniofacial anomalies associated with a genetic disease, i.e., Pierre Robin sequence).

The main orthodontic issues and so treatment goals for the two phenotypes of OSAS pediatric subjects are reported in Table 2.

All the previously described classifications should be considered not only for their academic value but also for their clinically oriented treatment choice potential. The multidisciplinary approach to identify OSAS child comorbidities and risk factors in their complexity is necessary to start the correct treatment plan.

### 2.1. Orthodontic Therapy Perspectives

The pediatric dentist can contribute to the treatment of OSAS in children throughout the establishment of an orthodontic treatment plan. The known available orthodontic treatment options in OSAS pediatric subjects are as follows:

- Intraoral appliances (IOAs) mechanically working by hold and stabilization of the jaw in an anterior position and thus by the upper airway size increase.
- Rapid maxillary expansion (RME) orthopedically treating the maxillary contraction is possible in growing children. RME opens the median palatine suture and causes maxillary transverse expansion. It provides balanced occlusion, nasopharyngeal airway increase, and nasal resistance reduction, causing a facilitated nasal breathing. RME is also said to lead to a tongue anterior repositioning.
- Surgically assisted RME performed by maxillofacial surgeons and orthodontists in adult subjects, by surgical reopening of the median suture of the palate to obtain an orthopedic effect.
- Maxillomandibular advancement (MMA) maxillomandibular complex advancement in the sagittal plane can be performed surgically and it allows upper airway size and muscle tone increase (maxilla-facial relevance) [15].

Intraoral appliances (IOAs) are useful in the OSA treatment in the adult population and in the AHI improvement. They work by the mechanical jaw hold in an anterior position, not only affecting the anteroposterior dimension but also causing velopharynx lateral diameter increase. In growing subjects, the use of functional devices, able to stimulate the mandibular growth, can allow an improvement of OSAS-related symptoms. In mild or moderate OSA, IOAs can be used as an alternative to CPAP, while in severe OSA, they are employed when patients cannot tolerate CPAP.

Recent studies show controversial evidence about the effectiveness of the various treatment options of OSAS. In fact, some authors report that, from high-quality research, there is no evidence supporting RME, surgical or nonsurgical assisted, and/or MMA treatment in OSA patients [15].

Moreover, other authors report that long-term studies have shown that the long-term efficacy of these treatments is unsatisfactory, as they do not address the OSA etiological cause.

Furthermore, physical exercise has resulted in allowing a reduction in the severity of diseases and/or disorders OSAS-related, including cardiovascular disease, diabetes, obesity, and hypertension, which are both comorbidities and contributing causes of OSA. In fact, adipose tissue excess can induce airway collapse, causing episodes of apnea or hypopnea and the obesity itself is connected to the increased presence, in the pharyngeal airways, of fatty tissue [19].

From what concerns the pediatric OSA subject management, the orthodontic treatment aims to reduce its severity, increase the airspace, and improve the airflow. Orthodontic therapy options include orthopedic expansion of the upper jaw (RME) and mandibular advancement by intraoral appliances (IOAs). PSG results, after orthodontic treatment, suggest that it can allow an airway patency improvement.

Rapid maxillary expansion (RME) aims to determine an orthopedic effect upon the maxilla, improving in rebound the lingual position, increasing nasopharyngeal volume, stimulating maxillary complex growth, and increasing nasal breathing in children suffering from OSA. The success key in pediatric OSAS treatment lies in airway expansion. To gain long-term stability, the authors report being effective as an adjunctive treatment to adenotonsillectomy. Other RME advantages are as follows:

- AHI values improvements (5.79 events/hour average difference).
- Average oxygen saturation increase of 2.5%.
- Microawakening reduction of 2.17 events/hour (if caused by respiratory problems; excitation index, AI).
- Quality sleep improvement (REM phase sleep percentage increase).
- Sleep efficiency (SE) improvement [16].

The literature reports several studies analysing the RME treatment-induced airway improvements by the use of functional tests as rhinomanometry (diagnostic tool able to assess the nasal respiratory function) and acoustic rhinometry (“*new*” technique used to evaluate nasal obstruction by the record of a sound impulse introduced clinically by the nostrils). The records obtained by these diagnostic procedures indicate a significant nasal airway resistance reduction, gaining improved nasal breathing. Moreover, pharyngeal airway pressure decrease after RME treatment; this mechanism and the decrease in nasal resistance may cause a relief in OSAS symptoms in children [20, 21].

Mandibular advancement devices (MAD) aim to lower and advance the jaw, stimulating dilator muscles of the airways. MAD use effectiveness among OSAS treatment is justified by the effects they induce, as follows:

- Reduction in apnea/hypopnea values, with short-term improvement of the OSA parameters.
- Increase in velopharyngeal airway lateral dimension, as the result of the forwarded position of the mandible and the reduced airway flexibility.
- Dilator muscles stimulation (genioglossus) with improvement in the upper airway stabilization.
- Modification in the neuromuscular working on the craniofacial structure (both skeleton and dentition), producing skeletal growth and dentoalveolar changes.

And about the mandibular advancement itself, in general, the following was found:

- It improves upper airway patency during sleep, by both widening the upper airway and decreasing their tendency to collapse (obstruction prevention).
- It improves AHI in pediatric subjects, as a transitional treatment (i.e., preorthognathic postgrowth surgery) or in every-age patients not tolerating CPAP [22].

A key consideration in understanding whether MADs produce long-term stable effects or not is whether the post-MADs treatment PSG was performed with the device in situ. This aspect would clarify if the MAD use is able to produce skeletal changes or merely induce a temporary mandible forward repositioning when in situ. Since a forwarder inferior jaw position produces a widening in the oropharynx space, the OSAS parameters improvements may be the result of the MADs action of forward repositioning of the mandible and not of permanent improvement in craniofacial features [1].

A MAD-like effect can be obtained by the use of functional devices used in orthodontics to treat deep bite in class II patients, with better comfort and adherence to the treatment of the patients themselves. The greatest advantage of these functional devices lies in the possibility of combining orthodontic action and myofunctional treatment, achieving normal nasal ventilation and the balance between intra- and extraoral muscles. Adequate myofunctional reeducation in children allows long-term remission of OSAS symptoms, compared to OSAS subjects treated with RME and/or adenotonsillectomy without the myofunctional reeducational component. The main limit of the myofunctional therapy is the difficulty that growing patients go through to perform correctly and consistently the exercises if under the age of 4.

The myofunctional therapy (MFT), previously introduced, has to be conducted by a speech therapist, in which collaboration makes a great advantage in OSAS treatment if performed as combined with the orthodontic therapy. One of the aims of the MFT is the soft palate elevation, which recruits different upper airway muscles (tensor and palatine veil elevator, palatoglossus, palatopharyngeal, elevators of the mandible, and tongue muscles). The mechanism of the MFT is based on muscle training, with specific muscular coordination, endurance, and tone improvements, considering the specificity of every single exercise (if isometric or isotonic). MFT exercises improve muscular fatigue in OSAS patients and work on the balance between the muscles acting in different pharyngeal segments (oropharyngeal, velopharyngeal, and hypopharyngeal). Moreover, MFT can reduce volume and fat in pharynx structure and muscles, thereby contributing to the decrease of upper airway collapsing episodes in OSA subjects [23].

MFT exercises involve oral (lips and tongue) and extraoral structures (soft palate and lateral pharyngeal wall). Every muscle or structure training produces different and specific effects:

- Soft palate: oral vowel sound pronunciation, continuously (with isometric exercises) or intermittently (with isotonic exercises).
- Tongue: moving it upon upper and lateral teeth surfaces, placing the tongue tip against the hard palate, pushing entirely the tongue against the soft and hard palate, or forcing it to the mouth floor.
- Face: by orbicular muscle movements of contraction and relaxation and lateral movements of buccinators and mandible muscles.
- Stomatognathic functions: inhaling nasally and exhaling orally, together with specific swallowing/chewing training exercises [24].

MFT, according to multiple studies, provides an adjuvant treatment in OSA therapy, with oxygen saturation increase and orofacial complex myofunctional state improvement. MFT can also reduce the following:

- AHI by 50% in adults and 62% in children.
- Sleepiness and snoring, with positive results at follow-up compared to controls [24].

## 3. Conclusions and Recommendations

Sleep-disordered breathing (SDB) is commonplace during childhood, with Obstructive Sleep Apnea Syndrome (OSAS) representing its most severe consequence. Obstructive apnea episodes are a relevant health problem, caused by the upper airway collapse during sleep and involving the reduction of the cessation in the airflow (hypopnea or apnea), the establishment of atypical sleep patterns (sleep fragmentation), and blood-gas level alteration (oxygen desaturation), with child quality of life damage. In children, every apneic episode must be considered pathological. OSA patients show a wide variability of breathing disorders in-sleep manifestations, clinically resulting in diurnal and nocturnal symptoms. Nocturnal symptoms include habitual snoring, dry mouth, forced oral breathing, abnormal thoracic and/or abdominal movements, enuresis, restless sleep with breathing pauses, awakenings and position changes, and sweating. Diurnal symptoms include nasal breathing difficulties, morning headache, hyperactivity and/or irritability, poor school/academic performance, sleepiness (more frequent in obese children or adolescents), stature development reduction, and cardiorespiratory complications. Primary snoring is the first SDB manifestation, and if not diagnosed or treated early, it can determine a progressive airway resistance increase, up to the OSAS occurrence. Therefore, in order to minimize the previously reported consequences, an early correct diagnosis is key. Due to its clinical features' complexity, OSAS management requires a multidisciplinary approach by the plurispecialists team in order to make an early diagnosis and a correct treatment plan. Specialists deputized to treat and diagnose OSAS include pediatrician, otorhinolaryngologist, pediatric dentist, orthodontist, neurologist, nutritionist, physiotherapist, and speech therapist. The diagnosis itself is referred to the otorhinolaryngologist, but the pediatrician and the pediatric dentist play a key role in the early identification of clinical features of SDB in the children population. A pathological chronic protracted respiratory pattern in childhood is associated with skeleton and soft tissue anomalies, which identify a particular cephalometric stereotype and specific craniofacial features that the orthodontist is bound to observe and diagnose. In addition to the diagnostic role, the orthodontist and the pediatric dentist play a major role in OSAS treatment: scientific evidence shows that rapid maxillary expansion (RME) and mandibular advancement using intraoral appliances (IOAs) contribute to the improvement in childhood OSAS severity. Moreover, the myofunctional therapy (MFT), conducted by a speech therapist, in which collaboration makes a great advantage in OSAS treatment if performed as combined with the traditional orthodontic therapy, provides oxygen saturation increase and a myofunctional orofacial state improvement.

Nutritional education and physical exercise/training are part of the OSA therapeutic plan in children because obesity is one of the major risks and causative factors in the occurrence of apneic episodes and syndrome (with a greater role in adolescents and a less important role during childhood, when the main causative factor involves craniofacial growth alterations). The OSAS diagnostic flowchart begins with the submission of validated questionnaires to parents, goes through a clinical examination (ENT), and ends with the diagnostic confirmation by instrumental exams.

Dental examination is necessary to identify maxillary growth alteration, crossbite, dental crowding, increased overjet, and overbite. In case of habitual snoring or OSAS in a growing subject, with the concomitant presence of a correlated craniofacial morphology, the treatment with a rapid maxillary expander and/or mandibular advancement devices is necessary. In addition, subjects with OSAS-related craniofacial and occlusal morphology, associated with a history of snoring, asthma, inability to nasal breathing, allergies, and/or obesity, should be referred to the pediatrician for an evaluation and then to the ENT specialist for further instrumental examinations.

Scientific evidence shows how OSAS diagnostical and treatment management need a multidisciplinary approach: the orthodontic therapeutic approach in the management of the OSA episodes in the pediatric population includes orthopedic maxillary expansion and the mandibular advancement using intraoral appliances.

Hence, since the orthodontist and the pediatric dentist play an important role not only in early diagnosis but also in the treatment of pediatric OSAS, more specialist training and updating for the medical profession would be desirable as much as a bigger collaboration between specialists, in particular between orthodontists and otolaryngologists.

Despite the presence of many pathological-oriented classifications of OSAS children, in the literature, no clinically oriented treatment-making algorithm was performed. So, it would be desirable for future studies to elaborate a simplified scheme for treatment plan choice of pediatric OSAS able to include multidisciplinary parameters and risk factors such as age, comorbidities, craniofacial malformation, adenotonsillar hypertrophy, oral breathing, skeletal class, and presence and/or type of malocclusion. So, the pediatric dentist, as a sentinel for OSAS in children, could differentiate the clinical evaluation according to standard parameters, gaining the chance to start target therapies specifically for every patient.

## Figures and Tables

**Table 1:** Clinical manifestation of OSAS in the pediatric age: diurnal and nocturnal symptoms.

Nocturnal s.	(i) Habitual snoring
	(ii) Dry mouth
	(iii) Forced oral breathing
	(iv) Abnormal thoracic and/or abdominal movements
	(v) Enuresis
	(vi) Restless sleep with breathing pauses, awakenings, and position changes
	(vii) sweating

Diurnal s.	(i) Nasal breathing difficulties
	(ii) Morning headache
	(iii) Hyperactivity and/or irritability
	(iv) Poor school performance
	(v) Sleepiness (more frequent in obese children or adolescent)
	(vi) Stature development reduction
	(vii) Cardiorespiratory complications

**Table 2:** Greater orthodontic issues and goals in pediatric population OSAS subjects.

Orthodontic features	Orthodontic treatment
Increased mandibular angle	Orthopedic correction
Skeletal class II	Functional therapy and mandibular advancement
Increased overbite	Deep bite correction
Lower jaw retrusion	Mandibular correction
Retracted tongue	Myofunctional therapy

## Data Availability

The data supporting this review are from previously reported studies which have been cited.

## Abbreviations

SDB: Sleep-disordered breathing
OSAS: Obstructive Sleep Apnea Syndrome
UARS: Upper Airway Resistance Syndrome
RME: Rapid maxillary expansion
MADs: Mandibular advancement devices
IOA: Intraoral appliance.
